# Iodine-125 seed inhibits proliferation and promotes apoptosis of cholangiocarcinoma cells by inducing the ROS/p53 axis

**DOI:** 10.1007/s10142-024-01392-1

**Published:** 2024-06-12

**Authors:** Fuping Kang, Jing Wu, Li Hong, Peng Zhang, Jianjun Song

**Affiliations:** 1 Department of Hepatobiliary Surgery, General Hospital of Ningxia Medical University, 804 Shengli South Street, Yinchuan City, Ningxia Hui Autonomous Region 750004 China; 2 Medical Experiment Center, General Hospital of Ningxia Medical University, 804 Shengli South Street, Yinchuan City, Ningxia Hui Autonomous Region 750004 China; 3 Department of Pediatrics, People’s Hospital of Ningxia Hui Autonomous Region, Yinchuan City, Ningxia Hui Autonomous Region China

**Keywords:** Cholangiocarcinoma, Iodine-125 seed, ROS/p53 pathway, Proliferation, Apoptosis

## Abstract

With advances in radioactive particle implantation in clinical practice, Iodine-125 (^125^I) seed brachytherapy has emerged as a promising treatment for cholangiocarcinoma (CCA), showing good prognosis; however, the underlying molecular mechanism of the therapeutic effect of ^125^I seed is unclear. To study the effects of ^125^I seed on the proliferation and apoptosis of CCA cells. CCA cell lines, RBE and HCCC-9810, were treated with reactive oxygen species (ROS) scavenger acetylcysteine (NAC) or the p53 functional inhibitor, pifithrin-α hydrobromide (PFTα). Cell counting kit-8 (CCK-8) assay, 5-bromo-2-deoxy-uridine (BrdU) staining, and terminal deoxynucleotidyl transferase (TdT)-mediated dUTP nick end labeling (TUNEL) assay and flow cytometry assay were performed to test the radiation-sensitivity of ^125^I seed toward CCA cells at different radiation doses (0.4 mCi and 0.8 mCi). 2,7-dichlorofluorescein diacetate (DCF-DA) assay, real-time quantitative polymerase chain reaction (RT-qPCR), and western blot analysis were performed to assess the effect of ^125^I seed on the ROS/p53 axis. A dose-dependent inhibitory effect of ^125^I seeds on the proliferation of CCA cells was observed. The ^125^I seed promoted apoptosis of CCA cells and induced the activation of the ROS/p53 pathway in a dose-dependent manner. NAC or PFTα treatment effectively reversed the stimulatory effect of ^125^I seed on the proliferation of CCA cells. NAC or PFTα suppressed apoptosis and p53 protein expression induced by the ^125^I seed. ^125^I seed can inhibit cell growth mainly through the apoptotic pathway. The mechanism may involve the activation of p53 and its downstream apoptotic pathway by up-regulating the level of ROS in cells.

**Supplementary Information:**

The online version contains supplementary material available at 10.1007/s10142-024-01392-1.

## Introduction

Cholangiocarcinoma (CCA) develops from the epithelial cells of the bile duct and is classified based on its anatomical location into intrahepatic cholangiocarcinoma (iCCA), periportal cholangiocarcinoma (pCCA), and distal cholangiocarcinoma (dCCA). Recent studies have reported an increasing incidence of and mortality rate due to CCA (Cho et al. 2022). Given the aggressive nature of CCA and its tendency to be first diagnosed in advanced stages, treatment options are often limited. Radical resection, in particular, is often constrained in these cases (Laschos et al. 2020), and may also lead to serious postoperative complications, including hepatorenal syndrome and progressive liver failure (Zhu and Wei 2020). Therefore, it is particularly important to develop effective methods to inhibit the proliferation and invasion of CCA cells.

Interstitial radiotherapy, a form of radiotherapy, is less active than conventional radiotherapy in terms of radiation source activity, shorter treatment distance, and easier protection (Huang and O’Sullivan 2013). The radiation source can be directly implanted into tumor tissues, thus exerting a strong killing effect on tumor cells in the treatment target area and causing little damage to surrounding normal tissues (Zakeri et al. 2019). Iodine-125 seed (^125^I seed), as the main radioactive particles used in clinical settings in China, are small in size and low in energy (Wu et al. 2018) and can release large doses of γ-rays into tumor tissues at close ranges, which can inhibit the mitosis of tumor cells and significantly reduce the re-proliferation of tumor tissues, causing no damage to normal tissues with fewer toxic side effects (Zhang et al. 2020). In the 1970s, ^125^I seed brachytherapy was initially employed in prostate cancer (Zaorsky et al. 2017). At present, ^125^I seed implantation is extensively utilized in the clinical treatment of malignant tumors in the chest region (Jiang et al. 2021), head and neck (Zhang et al. 2022), abdomen (Sugawara et al. 2011), and soft tissues (Chen et al. 2022a, b). After a long-term follow-up observation of patients receiving ^125^I seed implantation treatment, the functions of the urinary system, reproductive system, and rectal system of patients were found to be well protected and maintained in a previous study (Buckstein et al. 2013). The ^125^I seed can enhance the prognosis of patients who have experienced recurrence or failure of radiation therapy. Two recent clinical studies have demonstrated the therapeutic value of the ^125^I seed in CCA (Luo et al. 2022; Chen et al. 2022a, b). However, unlike the widespread clinical development, research on the molecular mechanism underlying the anti-tumor effect of ^125^I seed implantation is relatively lacking. Previous studies have identified inhibition of proliferation and apoptosis induction as potential antitumor mechanisms underlying ^125^I seed brachytherapy (Zhang et al. 2016; Zhuang et al. 2009). However, comprehensive evidence on this topic, especially at the molecular level, is lacking.

Reactive oxygen species (ROS), including superoxide anions, hydrogen peroxide, and hydroxyl radicals, are byproducts of normal cellular metabolism (Cui et al. 2022), . Moderate ROS production is involved in regulating signal transduction and gene expression in normal cells, thus maintaining the cellular physiological balance. However, high ROS level can directly or indirectly cause apoptosis of cells. Directly, ROS can damage cellular nucleic acids, proteins, and lipids, eventually disrupting the biological molecular structure and function of cells. Indirectly, ROS can mediate multiple key apoptosis signaling pathways, such as regulating the release of cytochrome c, activating the cysteine protease family, and activating caspase, thereby accelerating the apoptosis of cells (Barbieri and Sestili 2012). Furthermore, compelling evidence suggests that the ^125^I seed significantly elevates ROS level in tumor cells, in turn inducing apoptosis (Liu et al. 2018) but whether ^125^I seed in CCA regulates proliferation and apoptosis through ROS signaling remains unclear.

p53 is induced by ROS and regulates the proliferation and apoptosis of tumor cells (Cao et al. 2020; Xing et al. 2022; Shi et al. 2020), and both p53 and ROS together inhibit the progression of CCA (Wandee et al. 2021; Ren et al. 2021). More importantly, p53 is induced by ^125^I seed in non-small lung cancer and colorectal cancer (Ma et al. 2014; Zhang et al. 2021). However, whether the ^125^I seed in CCA can promote p53 expression to play an inhibitory role in cancer through up-regulation of ROS production is unknown.

This study focused on the effects of ^125^I seed treatment of different doses on CCA cells, and explored its possible regulatory mechanism, to provide experimental support for the development of new treatment options for CCA.

## Materials and methods

### Cell lines and culture

The CCA cell lines, RBE and HCCC-9810, were acquired from the National Biomedical Experimental Cell Resource Bank of China located in Beijing. RBE and HCCC-9810 cells were grown in RPMI-1640 medium supplemented with 10% fetal bovine serum (FBS). The cells were incubated at 37 ℃ in an incubator with 5% CO_2_. Specifically, 0.25% trypsin was used for passaging, and cells in the logarithmic growth phase were selected (after 48 h of culture) for experiments. In some experiments, cells were treated with 5 mmol/L NAC (Sigma)(Fan et al. 2020) or 100 nmol/L PFTα (Zhou et al. 2016).

### Irradiation of CCA cells with the ^125^I seed

^125^I seed for medical use was obtained from HTA Co., Ltd., Beijing, China. Experiments were conducted using the ^125^I seed at two different doses of 0.4 mCi and 0.8 mCi. RBE and HCCC-9810 cells were treated with various doses of the ^125^I seed for 72 h. Control cell lines were not irradiated with the ^125^I seed. We established an in vitro model of irradiation as follows: eight ^125^I seeds were evenly buried in a 30 mm diameter circular groove, and one was positioned at the center. In the experiment, a 6 cm culture dish was positioned in the ^125^I seed irradiation model within the chamber. RBE and HCCC-9810 cells (1 × 10^5^ cells per well) were inoculated in dishes with 6 cm diameter. To ensure equal irradiation, all dishes were periodically rotated clockwise at specific time intervals, as described previously (Zhang et al. 2021; Ren et al. 2022).

### Cell proliferation assay

Each group of cells was seeded into separate 96-well plates and incubated for different time intervals, i.e., 24, 48, and 72 h. Subsequently, each well of the 96-well plates was treated with 10 µL of the CCK-8 reagent (Dojindo Laboratories, Japan) and incubated at 37 °C for 2–3 h. The optical density values at 460 nm were measured using a Microplate Reader (Bio-Rad, USA)(Tian et al. 2015).

### Terminal deoxynucleotidyl transferase (TdT)-mediated dUTP nick end labeling (TUNEL) staining

Cells from each group were collected and adjusted to a final concentration of 1 × 10^5^ cells/ml. Promega’s DeadEnd™ Colorimetric TUNEL System was used to perform TUNEL staining as described previously (Hiraoka et al. 2013). Apoptotic cells were assessed by optical microscopy (Olympus, C5060-ADU, Tokyo, Japan), and the proportion of TUNEL-stained cells was calculated by randomly counting the cells in three separate fields of view.

### Flow Cytometry: apoptosis assay and cell cycle assay

In the cell apoptosis experiment, cells washed with PBS were collected and mixed with Annexin V and PI (5 mg/L) of 5 µL respectively. After incubation at 25 ℃ and dark for 15 min, 500 µL binding buffer was added and stood at 4 ℃ for 30 min. The apoptosis of cells was detected by flow cytometry (BD, Franklin Lakes, NJ, USA).

In a flow cytometry assay of the cell cycle, 1 × 10^6^ cells were initially collected and then re-suspended in pre-cooled 70% ethanol, where they were stored overnight at 4 °C. The following day, the cells were centrifuged, stained with PI after re-suspension, and incubated at 37 °C for 30 min. Subsequently, the cell cycle distribution was analyzed using flow cytometry.

### Reverse transcription-quantitative polymerase chain reaction (RTqPCR)

The TRIzol reagent (Invitrogen, Carlsbad, CA, United States) was used to extract total RNA from cultured cells. Reverse transcription was performed using a kit and real-time quantitative PCR analysis was conducted using the qPCR SYBR Green master mix dye method. The RNA was transcribed into cDNA before analysis. β-actin was the internal reference gene and was used for normalization. The relative expression level of the target gene was calculated using the 2^−△△Ct^ method (Pfaffl 2001). The primers used in the experiment are listed in Table 1.

**Table 1:** Primer sequences used in this study

Gene	Forward primer	Reverse primer
p53	AAAAGTCTAGAGCCACCGTCC	AGTCTGGCCAATCCAGGGAAG
p21	CCTGTCACTGTCTTGTACCCT	GCGTTTGGAGTGGTAGAAATCT
Bcl-2	GATAACGGAGGCTGGGATGC	TCACTTGTGGCCCAGATAGG
Bax	AAACTGGTGCTCAAGGCCC	CTTCAGTGACTCGGCCAGG
β-actin	CTGGAACGGTGAAGGTGACA	TGGGGTGGCTTTTAGGATGG

### Western blot analysis

Groups of cells were routinely lysed, denatured, subjected to protein quantification, electrophoresed, and transferred onto membranes as described previously (Zhuang et al. 2009). After blocking, the membranes were incubated with primary antibodies against Bax (1:1000, ab32503, Abcam, USA), Bcl-2 (1:1000, ab32124, Abcam, USA), p21 (1:1000, ab109520, Abcam, USA) and p53 (1:1000, ab32389, Abcam, USA) followed by incubation with rabbit anti-goat secondary antibody (1:5000, ab6741, Abcam, USA) at 25 ℃ for 1 h. After washing the membranes, the ECL reagent was used to detect the protein bands. The ImageJ software (version 1.45s, Wayne Rasband, National Institutes of Health, USA, public domain) was used for quantitative analysis of protein expression. β-actin (1:5000, ab8227, Abcam, USA) was used as a loading control.

### Measurement of intracellular ROS level

To measure intracellular ROS level, flow cytometry with the DCFH-DA probe was employed as described in a previous study(Tian et al. 2015). Cells were treated with 15 µmol/L PEITC for 6, 9, and 12 h, followed by incubation with 10 µmol/L of DCFH-DA probe, away from light for 15 min. After incubation, digested cells were resuspended in PBS and the fluorescence intensity of DCF was measured by flow cytometry (BD, Franklin Lakes, NJ, USA).

### 5-bromo-2-deoxyuridine (BrdU) assay

Following the instructions specified in the BrdU labeling and detection kit (Roche, Mannheim, Germany)(Lee et al. 2016), when the cells grew to a confluence of approximately 50%, the culture was supplemented with BrdU-labeled media and incubated for 15–60 min. The culture medium was discarded and cells were fixed overnight with 70% ethanol. The cells were incubated with the mouse anti-BrdU primary antibody (BD Biosciences, San Jose, CA, USA). Anti-mouse fluorescent antibody conjugated with FITC was the corresponding secondary antibody (Agilent Technologies, Santa Clara, California, USA).

### Statistical analysis

All experiments were performed in a triplicate. Data were analyzed using IBM’s SPSS 20.0 software. Measurement data are presented as mean ± standard deviation. One-way analysis of variance (ANOVA) was conducted to compare multiple groups. *P* < 0.05 indicated a statistically significant difference.

## Results

### Irradiation with the ^125^I seed inhibits the proliferation of CCA cells

**Fig. 1:**
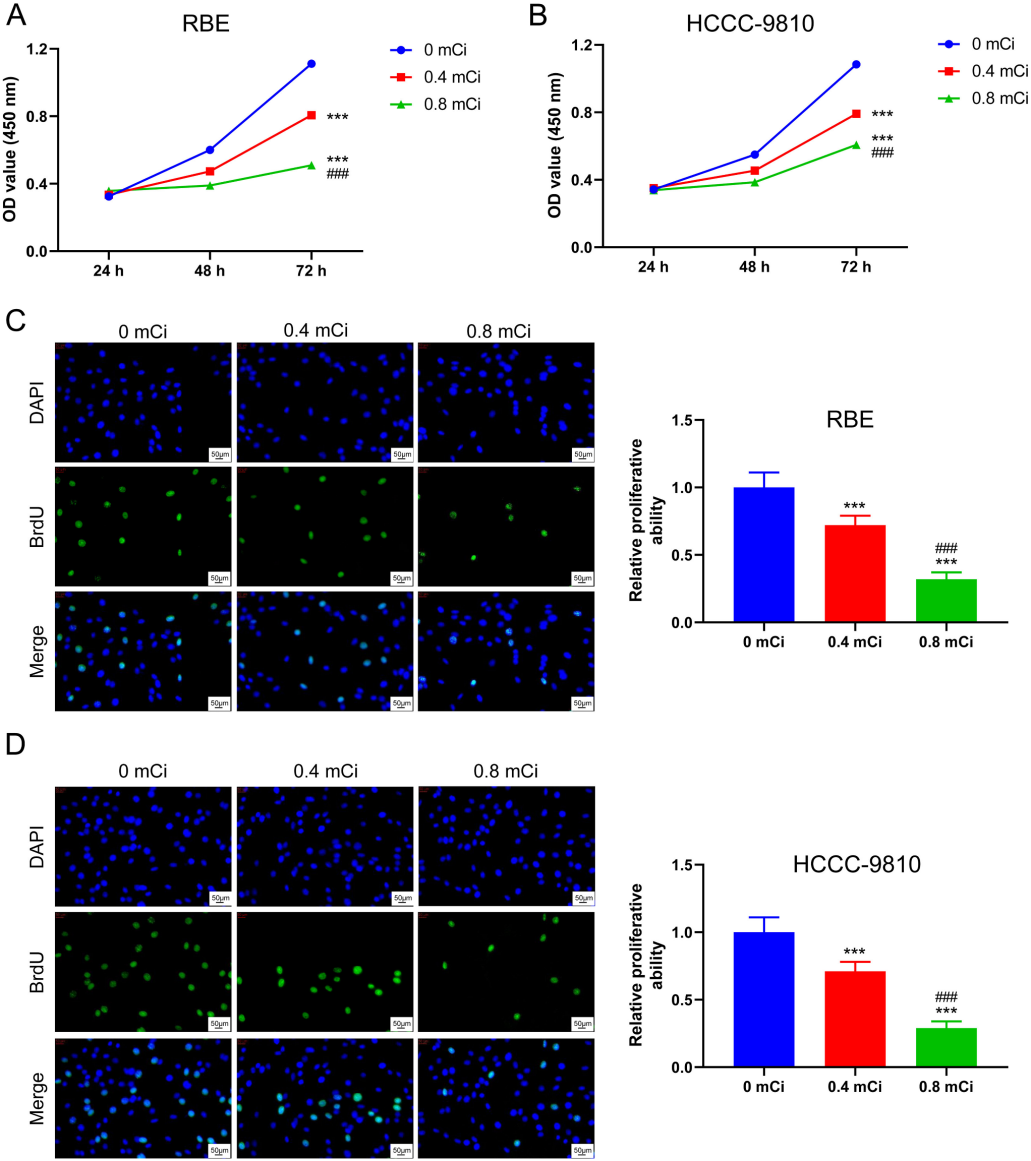
Irradiation with the^125^I seed inhibits the proliferation of CCA cells. CCK-8 assay indicated that ^125^I seed treatment gradually suppressed the viability of RBE (**A**) and HCCC-9810 cells (**B**). The proliferation of RBE (**C**) and HCCC-9810 cells (**D**) was gradually suppressed by ^125^I seed treatment, as indicated by the BrdU assay. ***P < 0.001 vs. 0 mCi group; ###*P* < 0.001 vs. 0.4 mCi group

We verified the effect of the ^125^I seed on the proliferation of CCA cell lines (RBE and HCCC-9810 cells) by the CCK-8 assay after continuous irradiation with 0, 0.4, and 0.8 mCi for 24 h, 48 h, and 72 h. The ^125^I seed significantly inhibited the viability of RBE and HCCC-9810 cells in a dose-dependent manner (*p* < 0.001, Fig. 1A **B**). To determine further whether the ^125^I seed inhibited the proliferation of CCA cells, BrdU assay was conducted and the results demonstrated that compared with the 0 mCi group, both 0.4 and 0.8 mCi ^125^I seed dosages inhibited the proliferation of RBE and HCCC-9810 cells, with the effect being prominent in the 0.8 mCi-treatment group (*p* < 0.001, Fig. 1C **D**). Taken together, the ^125^I seed negatively regulated the proliferation of CCA cells in a dose-dependent manner.

### The ^125^I seed induces apoptosis of CCA cells

**Fig. 2:**
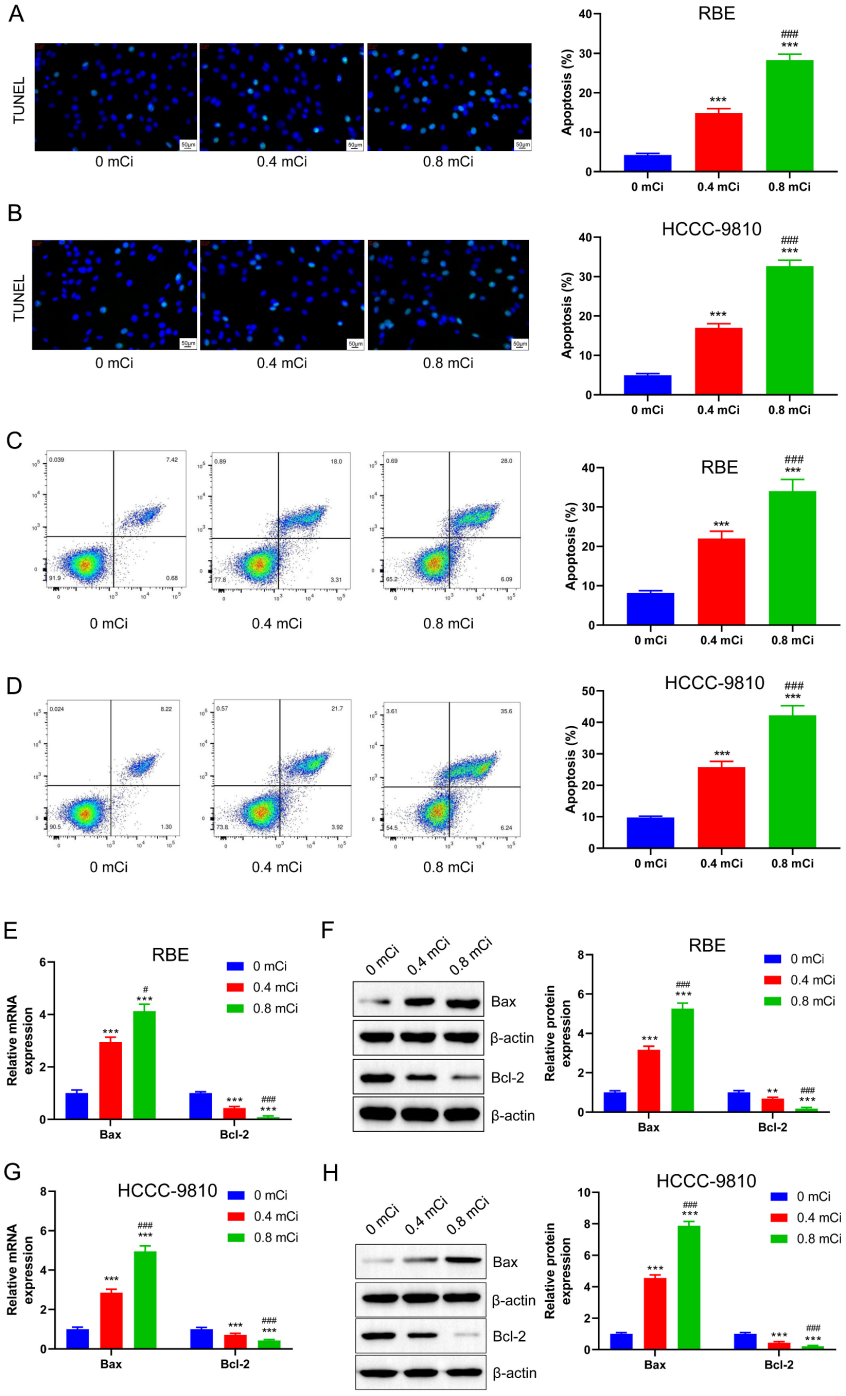
The^125^I seed induces apoptosis of CCA cells. TUNEL staining showed that ^125^I seed promoted apoptosis of RBE (**A**) and HCCC-9810 cells. Flow cytometry analysis indicated that the treatment of ^125^I seed for different doses induced apoptosis in RBE cells (**C**) and HCCC-9810 cells (**D**). RT-qPCR (**E**, **G**) and western blot analysis (**F**, **H**) showed that ^125^I seed promoted Bax expression and inhibited Bcl-2 expression in a dose-dependent manner in RBE and HCC-9810 cells. **P < 0.01, ***P < 0.001 vs. 0 mCi group; ###*P* < 0.001 vs. 0.4 mCi group

TUNEL staining after 72 h of ^125^I seed irradiation at doses of 0, 0.4, and 0.8 mCi showed that the ^125^I seed at doses of 0.4 and 0.8 mCi induced increased apoptosis in both RBE and HCCC-9810 cells, with the effect being more prominent in the 0.8 mCi group compared to 0.4 mCi group (*p* < 0.001, Fig. 2A **B**). The results of apoptosis measured by flow cytometry after 72 h of ^125^I seed irradiation at doses of 0,0.4 and 0.8 mCi showed that ^125^I seed at doses of 0.4 and 0.8 mCi induced increased apoptosis in both RBE and HCCC-9810 cells (*p* < 0.001, Fig. 2C **D**). Further, RT-qPCR analysis suggested that different doses of the ^125^I seed could promote Bax expression and simultaneously inhibit Bcl-2 expression in RBE cells. The effect on the 0.8 mCi group was more significant compared to the 0.4 mCi group (*p* < 0.05, *p* < 0.001, Fig. 2E). In RBE cells, western blot analysis showed that 0.4 and 0.8 mCi ^125^I seed treatment promoted the protein expression of Bax while inhibiting that of Bcl-2. The effect of ^125^I seed was more obvious in the 0.8 mCi group (*p* < 0.01, Fig. 2F). Simultaneously, ^125^I seed at both 0.4 and 0.8mCi doses promoted the mRNA expression of Bax, evidenced by RT-qPCR analysis, while inhibiting that of Bcl-2 in HCC-9810 cells (*p* < 0.001, Fig. 2G). Western blot showed in HCCC-9810 cells that with an increase in the ^125^I seed dose, the enhancing effect of the ^125^I seed on Bax and the inhibiting effect of Bcl-2 protein levels were more obvious (*p* < 0.001, Fig. 2H). Therefore, the ^125^I seed induced apoptosis in CCA cells, and the higher the ^125^I dose, the stronger the ability to induce apoptosis.

### ^125^I seed activates ROS/p53 pathway in CCA cells

**Fig. 3:**
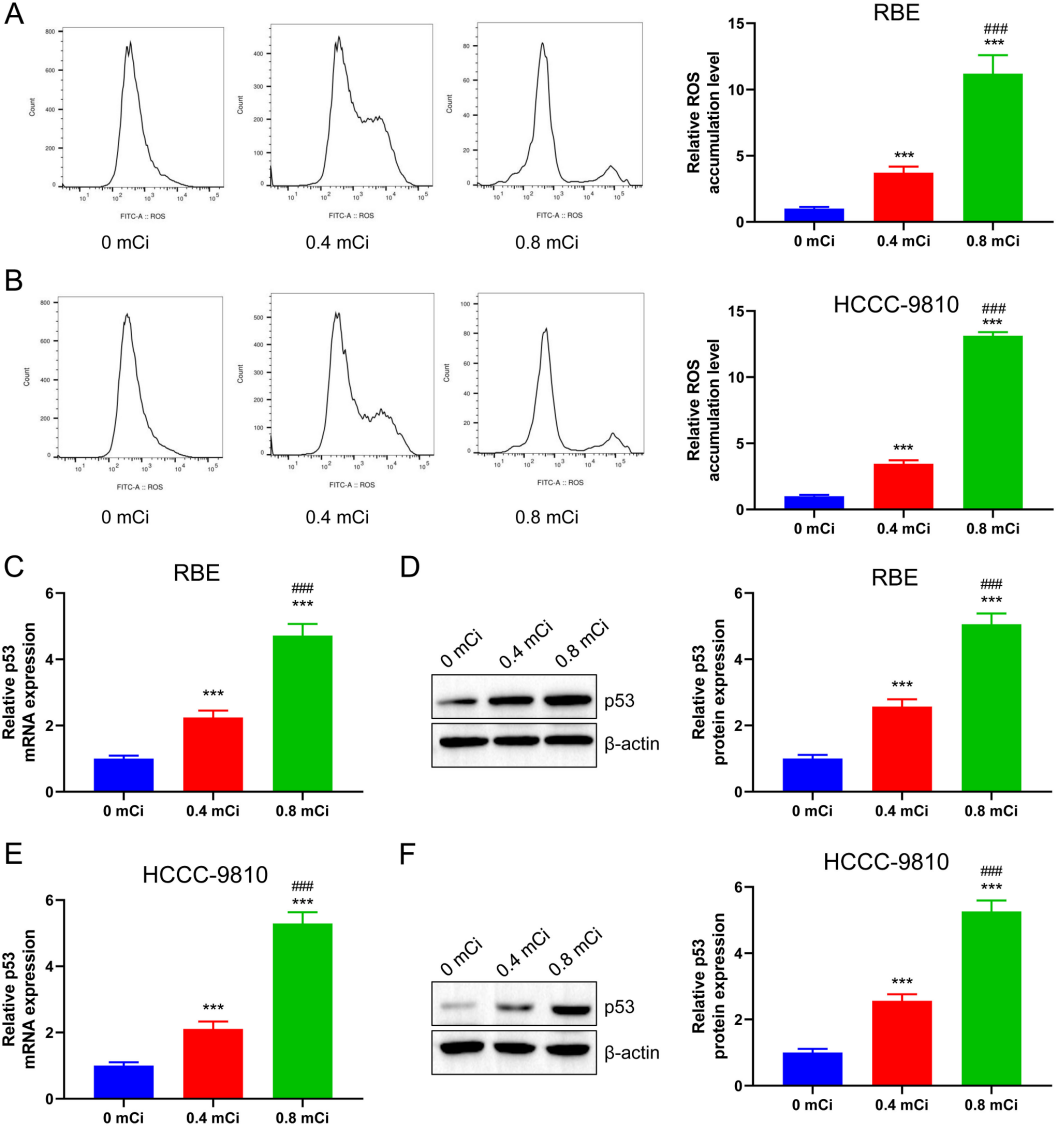
^125^I seed activates ROS/p53 pathway in CCA cells. The intracellular ROS levels of RBE cells (**A**) and HCCC-9810 cells (**B**) were increased after ^125^I seed treatment by flow cytometry. RT-qPCR (**C**, **E**) and western blot analysis (**D**, **F**) showed that ^125^I seed promoted p53 expression in RBE and HCC-9810 cells in a dose-dependent manner. ***P < 0.001 vs. 0 mCi group; ###*P* < 0.001 vs. 0.4 mCi group

The ^125^I seed induces increased ROS production (Wang et al. 2020) and p53 level (Xu et al. 2022). To further investigate whether the ^125^I seed in CCA plays a role in cancer suppression by upregulating ROS generation and promoting p53 expression, we first treated RBE and HCCC-9810 cells with 0.4 and 0.8 mCi doses of ^125^I seed for 72 h, respectively. Cells were subjected to DCFH-DA staining to detect ROS production. ROS level in RBE and HCCC-9810 cells of the 0 mCi group were low and increased significantly after treatment with the ^125^I seed (0.4 and 0.8 mCi). ROS level tended to increase with higher doses of the ^125^I seed (*p* < 0.001, Fig. 3A **B**). To determine whether ^125^I seed induced p53 expression in CCA cells, we detected p53 level in RBE cells by RT-qPCR. p53 level increased remarkably after treatment with 0.4 and 0.8 mCi of the ^125^I seed (*p* < 0.001, Fig. 3C). Western blot analysis showed that treatment with 0.4 and 0.8 mCi ^125^I seed could promote the increase in p53 protein expression in RBE cells, and compared with the 0.4 mCi group, the induction of p53 expression was greater in the 0.8 mCi group (*p* < 0.001, Fig. 3D). Similar results were observed in HCCC-9810 cells (Fig. 3E **F)**. Both mRNA and protein levels of p53 increased in a dose-dependent manner following treatment with 0.4 and 0.8 mCi ^125^I seed in HCCC-9810 cells (*p* < 0.001). The above findings indicate that the ROS/p53 pathway regulates the proliferation and apoptosis of CCA cells following treatment with ^125^I seed.

### Inhibition of ROS generation blocks the^125^I seed-induced accumulation of ROS and upregulation of p53 in CCA cells

**Fig. 4:**
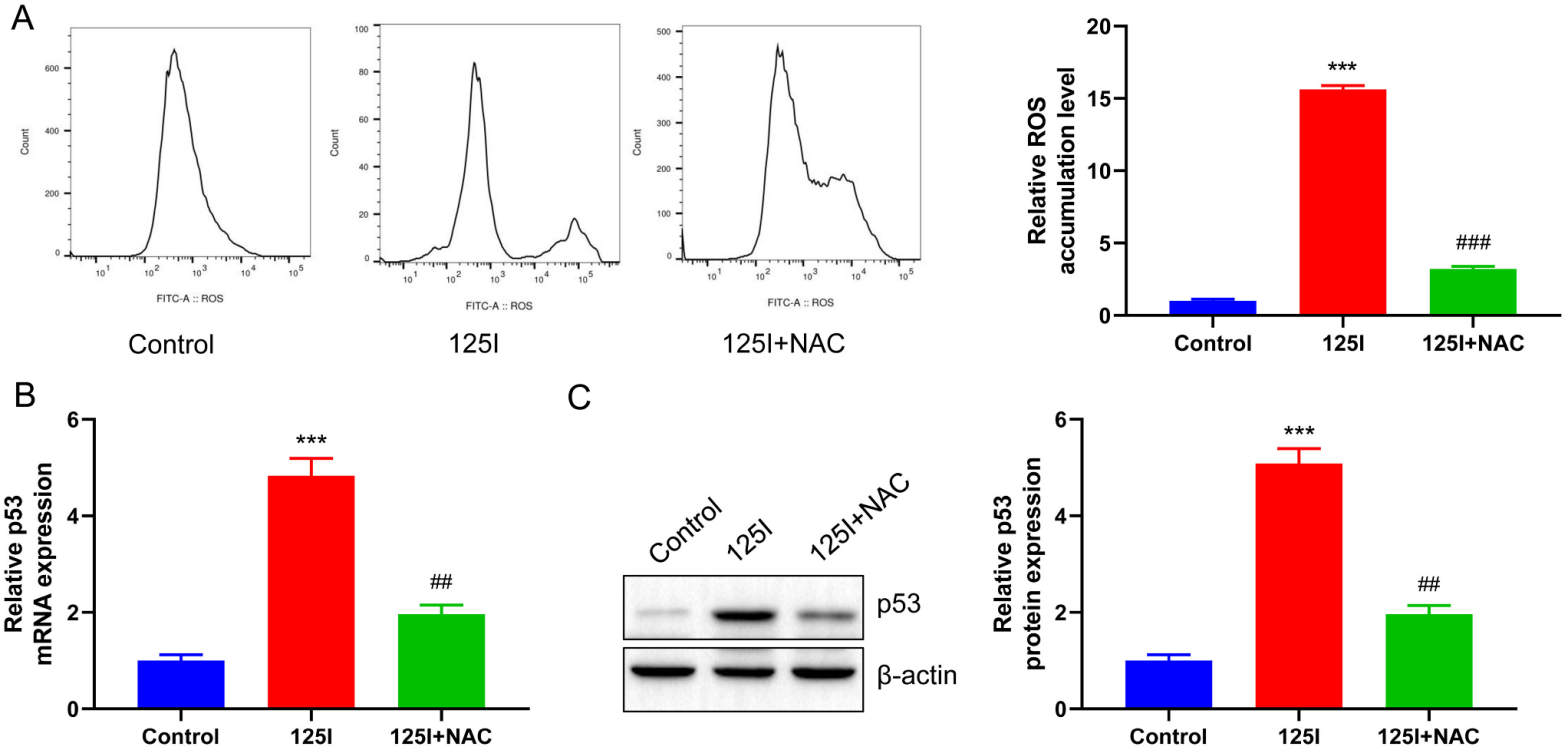
Inhibition of ROS generation blocks the^125^I seed-induced accumulation of ROS and upregulation of p53 in CCA cells. RBE cells were treated with 0.8 mCi of ^125^I seed and ROS scavenger NAC. (**A**) Flow cytometry assay indicated that NAC reduced intracellular ROS levels in RBE cells compared with ^125^I group. RT-qPCR (**B**) and western blot (**C**) analysis showed that NAC inhibited the mRNA and protein levels of p53 in RBE cells compared with ^125^I group. ***P < 0.001 vs. Control group; ##*P* < 0.01, ###*P* < 0.001 vs. ^125^I group

RBE cells were treated with 0.8 mCi of ^125^I seed and NAC, a scavenger of ROS, to study the effect of ROS on ^125^I seed-induced p53 upregulation. The DCFH-DA probe assay confirmed that the addition of NAC significantly reduced the increase in ROS level following treatment with 0.8 mCi ^125^I seed (*p* < 0.01, Fig. 4A). The mRNA level of p53, as detected by RT-qPCR, showed that NAC treatment could significantly inhibit the increase in p53 expression induced by the ^125^I seed (*p* < 0.01, Fig. 4B). Western blot analysis confirmed that the addition of NAC significantly inhibited ^125^I-induced increase in the protein level of p53 (*p* < 0.01, Fig. 4C). Collectively, inhibition of ROS generation effectively inhibited the accumulation of ROS and upregulation of p53 in RBE cells following ^125^I seed treatment.

### Inhibition of ROS production blocks the inhibitory effect of the^125^ I seed on the proliferation of CCA cells

**Fig. 5:**
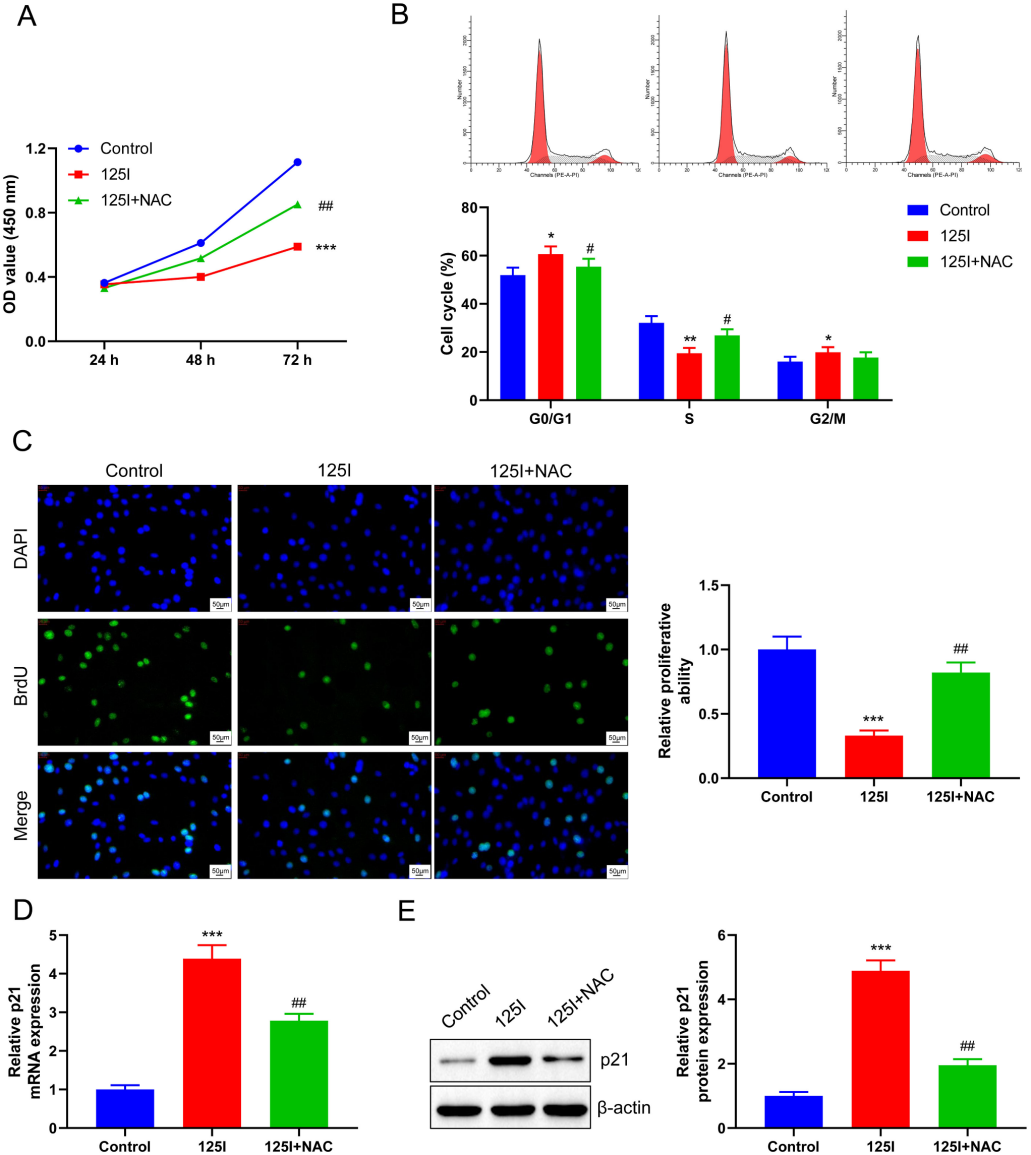
Inhibition of ROS production blocks the inhibitory effect of the^125^ I seed on the proliferation of CCA cells. RBE cells were treated with 0.8 mCi of ^125^I seed and ROS scavenger NAC. (**A**) CCK-8 assay showed that NAC promoted the viability of RBE cells compared with ^125^I group. (**B**) Flow cytometry showed that NAC blocked the inhibition of ^125^I seed on CCA cell cycle. (**C**) BrdU assay showed that the proliferation of RBE cells was increased by NAC compared with ^125^I group. RT-qPCR (**D**) and western blot analysis (**E**) showed that the mRNA and protein levels of p21 were significantly reduced in RBE cells after NAC treatment compared to the ^125^I group. *P < 0.05, **P < 0.01,***P < 0.001 vs. Control group; #*P* < 0.05, ##*P* < 0.01 vs. ^125^I group

Results of the CCK-8 assay demonstrated that cell viability decreased significantly after ^125^I seed treatment compared with the control, while NAC could rescue the inhibitory effect of ^125^I seed on the proliferation of RBE cells to some extent compared with the ^125^I group (*p* < 0.01, Fig. 5A). Next, flow cytometry analysis further demonstrated that ^125^I seed inhibition of cell cycle progression was the reason for its anti-cell proliferation effect, and NAC could reduce this effect of ^125^I seed. As shown in Fig. 5B, RBE cells showed different cell cycle distribution patterns under different treatments. Compared with the control group, ^125^I seed induced G0/G1 and G2/M cycle arrest and a decrease in the number of S-phase cells, while, as expected, adding NAC to ^125^I seed pretreated RBE cells resulted in a decrease in the number of G0/G1 phase cells and an increase in the number of S-phase cells, but no significant differences were seen in G2/M phase cells. And the results of the BrdU assay showed that NAC rescued the inhibitory effect of the ^125^I seed on the proliferation of RBE cells to some extent (*p* < 0.01, Fig. 5C). p21, a key player in p53-mediated growth inhibition, is a tumor suppressor (Maheshwari et al. 2022). We next confirmed through RT-qPCR that the increase in p21 level induced by ^125^I seed was reduced to a certain extent following the inhibition of ROS production (*p* < 0.01, Fig. 5D). Western blot analysis showed that the ^125^I seed could significantly induce the expression of p21 protein, while NAC could partially rescue this effect (*p* < 0.01, Fig. 5E). Thus, our findings support that the inhibition of ROS rescues the inhibitory effect of ^125^I seed on cell proliferation.

### Inhibition of ROS production blocks the promoting effect of^125^I seed on the apoptosis of CCA cells

**Fig. 6:**
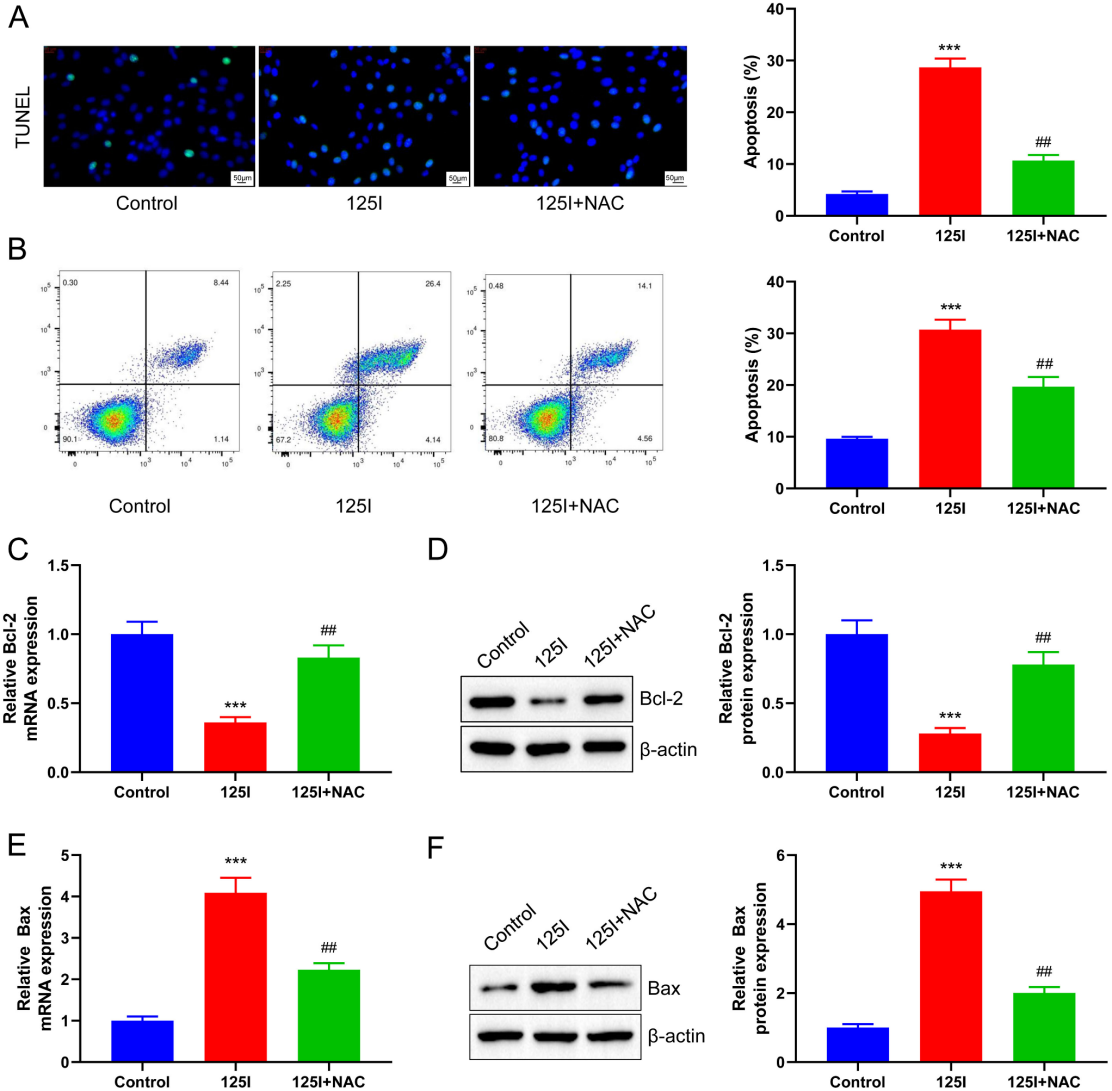
Inhibition of ROS production blocks the promoting effect of^125^I seed on the apoptosis of CCA cells. RBE cells were treated with 0.8 mCi of ^125^I seed and ROS scavenger NAC. (**A**) TUNEL staining showed that NAC inhibited RBE cell apoptosis compared with that in the ^125^I group. (**B**) Flow cytometry showed that NAC inhibited RBE cell apoptosis induced by ^125^I seed. RT-qPCR (**C**) and western blot analysis (**D**) showed that the mRNA and protein levels of Bcl-2 in RBE cells were promoted by NAC compared with ^125^I seed group. RT-qPCR (**E**) and western blot analysis (**F**) showed that the mRNA and protein levels of Bax in RBE cells were inhibited by NAC compared with ^125^I seed group. ***P < 0.001 vs. Control group; ##*P* < 0.01 vs. ^125^I group

Subsequently, TUNEL staining and flow cytometry were performed to detect whether NAC could affect the induction of apoptosis by ^125^I seed on RBE cells. Apoptosis of RBE cells treated with 0.8 mCi seed increased remarkably (*p* < 0.001), and NAC could effectively inhibit this effect (*p* < 0.01, Fig. 6A **B**). RT-qPCR and western blot analysis were performed to detect the mRNA and protein expressions, respectively, of apoptosis-related proteins, Bcl-2 and Bax. Treatment of RBE cells by NAC induced by the ^125^I seed significantly inhibited the decrease in Bcl-2 induced by the ^125^I seed (*p* < 0.01, Fig. 6C **D**) and remarkably inhibited the increase in the expression of the pro-apoptotic protein, Bax, induced by the ^125^I seed (*p* < 0.01, Fig. 6E **F**). In conclusion, inhibition of ROS production effectively inhibited cellular apoptosis induced by the ^125^I seed.

### Blockade of p53 attenuates the effect of the^125^I seed on the proliferation and apoptosis of CCA cells

**Fig. 7:**
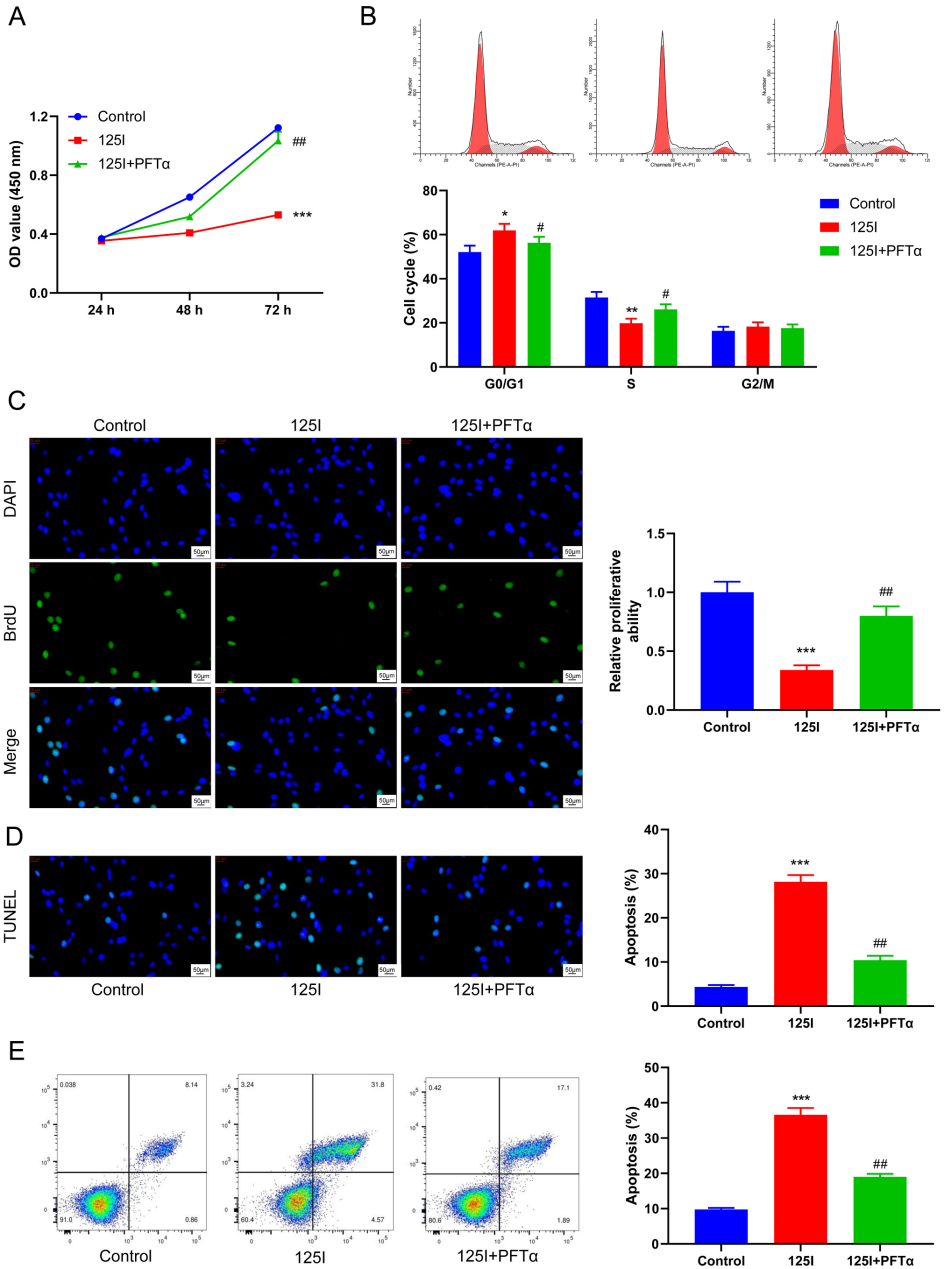
Blockade of p53 attenuates the effect of the^125^I seed on the proliferation and apoptosis of CCA cells. RBE cells were treated with 0.8 mCi of ^125^I seed and PFTα, a functional inhibitor of p53. (**A**) CCK-8 assay indicated that PFTα could rescue the inhibitory effect of ^125^I seeds on RBE cell proliferation. (**B**) Flow cytometry showed that PFTα blocked the inhibition of ^125^I seed on CCA cell cycle. (**C**) BrdU assay showed that the proliferation of RBE cells was increased by PFTα compared with ^125^I group. (**D**) TUNEL staining and Flow cytometry analysis (**E**) showed that the apoptosis of RBE cells was inhibited by PFTα compared to ^125^I group. *P < 0.05, **P < 0.01,***P < 0.001 vs. Control group; #*P* < 0.05, ##*P* < 0.01 vs. ^125^I group

Finally, ^125^I seed-induced RBE cells were treated with PFTα, a functional inhibitor of p53. Both CCK-8 and BrdU assays demonstrated that PFTα significantly rescued the inhibitory effect of ^125^I seed on the viability and proliferation ability of RBE cells (*p* < 0.01, Fig. 7A **C**). To further elucidate the regulatory role of p53 in cell proliferation, we analyzed the cell cycle distribution of RBE cells using PI staining combined with flow cytometry. Flow cytometry showed that PFTα partially reversed ^125^I-induced increase in the number of G0/G1 phase cells and decrease in the number of S phase cells compared with 125I group, but there was no significant change in G2/M phase cells in each group (*p* < 0.05, Fig. 7B**)**. And results of the TUNEL staining and flow cytometry assay revealed that ^125^I seed-induced apoptosis in RBE cells was reduced following treatment with PFTα (*p* < 0.01, Fig. 7D **E**). Thus, PFTα could significantly affect the regulatory effect of ^125^I seed on the proliferation of CCA cells.

## Discussion

Interventional chemoembolization or chemoinfusion were the main treatment options for inoperable progressive CCA (Bramlet et al. 1983), however, CCA is a tumor primarily without blood supply. Interventional chemoembolization or chemoinfusion thus fail to significantly improve the survival of these patients (Sahara et al. 2012). ^125^I seed implantation brachytherapy has been proven to be effective in various cancers (Kobayashi et al. 2013; Moon et al. 2012; Reddi et al. 2012).

^125^I seed implantation is a low-dose, brachytherapy treatment. Radiotherapy, especially implanted particles in CCA cells, could partially improve prognosis and provide effective relief for tumor pain (Kuhn et al. 2002). Ma et al. verified that the effective inhibition dosage of ^125^I seed ranged from 0.4 to 0.8 mCi (Ma et al. 2014). The 0.4 and 0.8 mCi doses of ^125^I seed effectively inhibited the proliferation of CCA cells (Fig. 1), consistent with previously reported findings (Ma et al. 2014). However, Moon et al. concluded that CCA cells were radioresistant (Moon et al. 1997). The reason for this difference may be different doses of radiation therapy used across studies. Accumulating evidence has shown that a mechanism by which ^125^I seeds treat tumors is through releasing γ-rays, which damage the DNA double strands in tumor cells and induce apoptosis(Xie et al. 2015). ^125^I seed treatment can modulate the biological function of CCA cells, including apoptosis induction and cell cycle arrest (Zhou et al. 2022). In this study, cells were grown for 72 h post-^125^I seed treatment to facilitate a meaningful comparison with previous findings (Ren et al. 2022). The ^125^I seed exerted a dose-dependent effect on inhibiting proliferation and promoting apoptosis in RBE and HCCC-9810 cells (Figs. 1 and 2). However, we must acknowledge certain adverse effects of ^125^I seed implantation brachytherapy exist (Almeida et al. 2011; Grewal et al. 2009). While the reported risk of persistent side effects due to ^125^I seed therapy is relatively small, data suggest that extreme caution needs to be exercised when selecting patients to administer ^125^I seed therapy(Ma et al. 2014). For patients with CCA with a moderate-to-high risk of recurrence, the smallest effective dose of ^125^I seed should be administered to maximize potential benefits and minimize the risk of adverse events. Herein, both 0.4 and 0.8 mCi ^125^I seed dosages inhibited the proliferation of CCA but given the side effects of the ^125^I seed, 0.4 mCi dose may be preferred for subsequent in vivo experiments and clinical trials.

Mitochondria are key determinants of cell fate. Normal cells generally maintain low- to-moderate level of ROS, whereas tumor cells, by upregulating ROS level, cause organelle damage, ultimately inducing cell death (Lu et al. 2019). Accumulating evidence has shown that radiation therapy can elicit cancer cells to produce excess ROS (Tian et al. 2015; Hein et al. 2014; Leach et al. 2001). Hu et al. showed that ^125^I seed irradiation caused mitochondrial damage, leading to a marked increase in intracellular ROS level in HCT116 cells (Hu et al. 2016). Therefore, in this study, the role of ROS in ^125^I seed-induced apoptosis of CCA cells was determined. ROS level increased in a dose-dependent manner after ^125^I seed treatment (Fig. 3A B). Further experiments on the effects of ROS showed that pre-treatment with a ROS scavenger (NAC) blocked the inhibition by ^125^I seeds on the proliferation and the induction of apoptosis of CCA cells (Figs. 5 and 6), indicating that ROS is involved in the inhibition of growth and early apoptosis of CCA cells.

p53 is an important downstream regulator of ROS-induced apoptosis (Arnandis et al. 2018). Many studies have shown that ROS production can lead to DNA damage, in turn activating the ATM/ATR-p53 signaling pathway (Park et al. 2021). To confirm whether the ^125^I seed treatment was dependent on the ROS-p53 pathway for induction of apoptosis, RT-qPCR and western blot showed that ^125^I seed treatment could significantly induce protein expression of p53 compared to the control (Fig. 3C, D, E, F). Intracellular p53 protein level decreased significantly after pretreatment with NAC (Fig. 4), suggesting that ROS played a crucial role in inducing the upregulation of p53 expression. As an important intracellular transcription factor, p53 can directly regulate the expression of its downstream apoptosis-related gene, Bax (Li et al. 2022). Bax level were significantly downregulated after NAC treatment, while those of Bcl-2 were significantly upregulated (Fig. 6C, D, E, F). The ^125^I seed can induce apoptosis in CCA cells by antagonizing its anti-apoptotic effect through the formation of heterodimers of Bax and Bcl-2. Therefore, we speculated that the pro-apoptotic mechanism of ^125^I seed may be realized by up-regulating intracellular ROS production, promoting p53 and Bax expression, and inhibiting Bcl-2 level. In addition, we also found that ^125^I seed promoted the expression of p21 and induced RBE cells to increase in G0/G1 cells while decreasing in S phase. Therefore, we speculated that ^125^I seed may also promote p21 through P53, thereby keeping cells in G1 phase and shortening S phase, thus inhibiting cell proliferation. However, it should be noted that normally, G1 phase could repair damaged DNA, but this study could not rely on blocking G1 phase to repair damaged DNA, because ^125^I seed can also cause oxidative stress and other damage, thus inhibiting cell proliferation and inducing apoptosis (Fig. 5). The role of p53 expressional upregulation in mediating the effect of ^125^I seed treatment on the biological functions of CCA cells is unclear. Therefore, PFTα, a functional inhibitor of p53, was used to suppress p53 level induced by ^125^I seed treatment. We analyzed the proliferation and apoptosis of CCA cells in the PFTα pre-treatment group, which increased significantly after ^125^I seed treatment, while apoptosis was significantly decreased (Fig. 7). Taken together, our findings suggest that the regulation of ^125^I seed’s effects on CCA cells depends on the p53 level.

However, it is worth noting that no studies have shown p53 mutations in the CCA cell lines used in this study (RBE and HCC-9810 cell lines), so this manuscript has certain limitations. Next, we will continue to explore the effects of ^125^I seed on CCA cells carrying p53 mutations.

## Conclusion

In summary, ^125^I seed inhibited cell growth through the apoptotic pathway. The mechanism may involve the activation of p53 and its downstream apoptotic processes by up-regulating ROS production in cells.

### Electronic supplementary material

Below is the link to the electronic supplementary material.

Supplementary Material 1

## Data Availability

The datasets generated during and/or analysed during the current study are available from the corresponding author on reasonable request.
