# Supplementary material for: Iodine-125 seed inhibits proliferation and promotes apoptosis of cholangiocarcinoma cells by inducing the ROS/p53 axis
Source: Funct Integr Genomics. 2024 Jun 12;24(3):114. doi: 10.1007/s10142-024-01392-1 (PMC11166828; doi:10.1007/s10142-024-01392-1)
Supplement: Supplementary file 1 — Supplementary Material 1 [file 10142_2024_1392_MOESM1_ESM.docx]

**Repeat one**

**Figure 2**

**F**

**

**

**Bax**

**

**

**Bcl-2**

**H**

**

**

**Bax**

**

**

**Bcl-2**

**Figure 3**

**D**

**

**

**p53**

**F**

**

**

**p53**

**Figure 4**

**C**

**

**

**p53**

**Figure 5**

**E**

**

**

**P21**

**Figure 6**

**D**

**

**

**Bcl-2**

**F**

**

**

**Bax**

**Repeat two**

**Figure 2**

**F**

**

**

**Bax**

**

**

**Bcl-2**

**H**

**

**

**Bax**

**

**

**Bcl-2**

**Figure 3**

**D**

**

**

**p53**

**F**

**

**

**p53**

**Figure 4**

**C**

**

**

**p53**

**Figure 5**

**E**

**

**

**P21**

**Figure 6**

**D**

**

**

**Bcl-2**

**F**

**

**

**Bax**

**Repeat three**

**Figure 2**

**F**

**

**

**Bax**

**

**

**Bcl-2**

**H**

**

**

**Bax**

**

**

**Bcl-2**

**Figure 3**

**D**

**

**

**p53**

**F**

**

**

**p53**

**Figure 4**

**C**

**

**

**p53**

**Figure 5**

**E**

**

**

**P21**

**Figure 6**

**D**

**

**

**Bcl-2**

**F**

**

**

**Bax**
